# Biological Roles of Protein-Coding Tandem Repeats in the Yeast *Candida Albicans*

**DOI:** 10.3390/jof4030078

**Published:** 2018-06-29

**Authors:** Matt Wilkins, Ningxin Zhang, Jan Schmid

**Affiliations:** 1 Centre for Professional and Continuing Education, Massey University, Palmerston North 4412, New Zealand; M.C.Wilkins@massey.ac.nz; 2 Institute of Fundamental Sciences, Massey University, Palmerston North 4412, New Zealand; N.X.Zhang@massey.ac.nz

**Keywords:** protein coding DNA tandem repeats, mutation rate, proteome evolution, amino acid tandem repeats, *Candida albicans*, synonymous mutations, Red Queen’s race, contingency genes

## Abstract

Tandem repeat (TR) DNA mutates faster than other DNA by insertion and deletion of repeats. Large parts of eukaryotic proteomes are encoded by ORFs containing protein-coding TRs (TR-ORFs, pcTRs) with largely unknown biological consequences. We explored these in the yeast *Candida albicans*, an opportunistic human pathogen. We found that almost half of *C. albicans’* proteins are encoded by TR-ORFs. pcTR frequency differed only moderately between different gene (GO) categories. Bioinformatic predictions of genome-wide mutation rates and clade-specific differences in pcTR allele frequencies indicated that pcTRs (i) significantly increase the genome-wide mutation rate; (ii) significantly impact on fitness and (iii) allow the evolution of selectively advantageous clade-specific protein variants. Synonymous mutations reduced the repetitiveness of many amino acid repeat-encoding pcTRs. A survey, in 58 strains, revealed that in some pcTR regions in which repetitiveness was not significantly diminished by synonymous mutations the habitat predicted which alleles were present, suggesting roles of pcTR mutation in short-term adaptation and pathogenesis. In *C. albicans* pcTR mutation apparently is an important mechanism for mutational advance and possibly also rapid adaptation, with synonymous mutations providing a mechanism for adjusting mutation rates of individual pcTRs. Analyses of *Arabidopsis* and human pcTRs showed that the latter also occurs in other eukaryotes.

## 1. Introduction

Tandem DNA repeats (TRs) are common in protein-coding regions of eukaryotic genomes [1]. The length of the repeated unit varies greatly, from a single base to hundreds of bases (units of <10 bases are commonly referred as microsatellites, longer units as minisatellites) [1]. TR regions are believed to arise by insertions of transposable elements or by replication slippage. Under the latter scenario, TR regions arise in an area between two non-contiguous copies of the same short sequence: the transient disassociation of DNA polymerase after replicating one copy and its re-joining at the other copy leads to a duplication of the region in between. Subsequently the number of copies of the duplicated region increases further by replication slippage in the duplicated region, or by homologous recombination [1,2,3,4].

Once established, TR regions mutate orders of magnitude faster than nonrepetitive DNA by insertion and deletion (indels) of repeat units, caused also by replication slippage and homologous recombination [5]. It is well established that indels of repeat units can have selectable phenotypic effects; examples include heritable disease in humans, changes in adhesion, alterations of regulatory networks and evasion of immune responses by pathogens [1,5,6,7,8]. For repeat regions that encode parts of proteins the alterations that cause such effects are by no means restricted to indel-induced frameshifts. Many protein-coding TRs (pcTRs) encode amino acid tandem repeats and indels will increase or decrease the length of these amino acid repeat regions. The effect on protein function may often be too subtle to be directly demonstrable [9,10] but may still lead to selectable fitness differences, in particular in microbial species. Because of their large effective population sizes, in microbial species even mutations with minute impact on fitness are under selection [11,12,13]. It is not uncommon for such series of encoded amino acid motifs to be conserved between species [14,15,16,17], an indication that they can confer selective advantages. In addition, a given habitat will often contain large numbers of microbial cells and this population will contain a pool of pcTR region-containing ORF (TR-ORF) variants, continually replenished by pcTR mutation. This provides an opportunity to use TR mutation for rapid adaptation if the habitat changes or to move to other habitats; that is, TR-ORFs can function as contingency genes [8,18].

The yeast *Candida albicans* is a frequent commensal colonizer of humans, mainly their gastrointestinal tract, and humans are apparently its primary habitat [19,20]. *C. albicans* can also cause both superficial and life-threatening systemic disease [19]. Its genome contains large numbers of pcTRs [21] and it is possible that pcTR mutation-generated protein variants could play a role in the transition from commensal to pathogen [16]. It is thus of interest to establish the biological role of pcTRs in this species and if such roles include roles in short-term adaptation. 

*C. albicans* has two features that also recommend it as a simple model for investigating the evolutionary role of pcTRs in general. One is that its mode of reproduction is almost exclusively clonal [22]. Thus TR-ORF allele combinations in the genome of a given strain are unlikely to result from a recent sexual recombination event but are likely to have coevolved. The second is the small number of ORFs containing introns [23]; as a result, disruption by introns should only have a minimal impact on the evolutionary biology of amino acid repeat-encoding TR regions. 

These reasons led us to undertake a genome wide survey of *C. albicans* TR-ORFs with the aim of elucidating the evolutionary consequences and role of pcTR mutation. 

## 2. Materials and Methods 

### 2.1. Analysis of Tandem Repeats in the C. albicans Genome

These analyses were based on FASTA files of coding sequences (CDS) and genomic gene sequences (including untranslated regions and introns), downloaded from the *Candida* Genome Database [24] (http://www.candidagenome.org; SC5314 assembly 22; accessed in April 2018).

For the analysis of TRs we wrote software (TRANALYSER) based on the SERV package designed by Legendre et al. [25]. SERV finds tandem repeat stretches using Tandem Repeats Finder [26] and for each stretch calculates a so-called VARscore. VARscores (i) predict the variability of a TR stretch, that is, the probability that when different individuals are assessed, their stretches will differ in length as a result of indels and (ii) provide an estimate of the frequency at which indels occur, that is, the rate of mutation (by insertions and deletions) of a TR-region [25]. 

**Figure 1:**
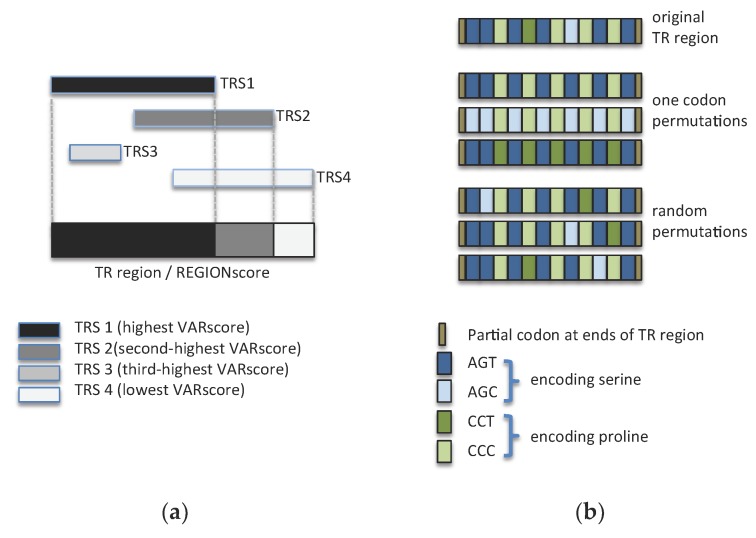
TRANALYZER processing of TR sequences (**a**) Determination of REGIONscores. REGIONscores are calculated by adding, to the VARscore of TR stretches (TRS) with higher VARscores, fractions of VARscores of lower-scoring stretches. The fraction of the score of the lower-scoring stretch added corresponds to the fraction of non-overlap with the higher scoring stretch. If an entire lower-scoring stretch is in a region covered by a higher-scoring stretch (such as TRS3), its VARscore is not considered; (**b**) Search for the maximum REGIONscore of a TR region encoding a series of serine/proline repeats by permutating it, without altering the amino acid sequence, by using either only one type of codon for each amino acid or by randomly shuffling codons.

Tandem Repeats Finder often reports multiple overlapping TR stretches for a given region of DNA, with longer stretches often corresponding to multiples of shorter stretches [26]. TRANALYZER combines such overlapping stretches into TR regions and uses a weighted sum of VARscores of TR stretches to calculate a VARscore for the entire TR region (REGIONscore). To do so, it first identifies the TR stretch with the highest VARscore (or several if they do not overlap) and then adds to it the VARscores of non-overlapping parts of other stretches with lower VARscores (Figure 1a). Because the non-overlapping parts of the other stretches may in themselves not represent a TR, the VARscore added is calculated as a fraction of the entire VARscore of these stretches, based on the degree of non-overlap. 

Mutation rate is exponentially related to VARscore and simply adding VARscores and fractions of VARscores to each other would therefore produce scores that suggest too high a differential between the mutation rate of a region and the mutation rate of its highest-scoring stretch. Therefore, before summing, VARscores are converted to mutation rate estimates (indels per generation; *ipg*) using the relationship between the two parameters experimentally determined in *Saccharomyces cerevisiae* [25]:

*ipg* = 5 × 10^−7^ × e^1.4×VARscore^ (1)

After the mutation rates have been added, *ipg*^−1^ can be applied to back-convert the sum back onto a VARscore for the entire region, that is, the REGIONscore. 

Thus, the REGIONscore can be defined as follows. For a region of length n, consisting of k stretches with individual VARscores $v_{i}$ and length $l_{i}$, i=1,…,k, the REGIONscore is:

(2) $$\;\mathrm{REGIONscore}=ipg^{-1}\left(\overset{n}{\underset{i=1}{\sum }}\frac{ipg\left(v_{m}\right)}{l_{m}}\right),$$

where $v_{m}$ is the maximum VARscore over those stretches defined at the ith base pair and $l_{m}$ is the corresponding length of that stretch. 

To estimate the maximum possible REGIONscore of a TR region encoding a given amino acid sequence, TRANALYZER generates permutations of the region in two ways (Figure 1b). The first is to use, for each amino acid, only one of the codons that is used, in the original TR-region, to encode that amino acid (one codon permutation). For instance, if in a TR region serine is encoded by AGT and AGC and proline in the region is encoded by CCT and CCC, then TRANALYZER generates four alternate TR regions in which only the codons AGT and CCT or only AGT and CCC or only AGC and CCT or only AGC and CCC are used. If there are more than 500 possible one codon permutations, then 500 are randomly picked for further analysis. The second approach (random permutation) involves switching the position of the existing codons in the sequence without altering the amino acid sequence encoded (or the number of times each of the codons is used; 100 such random permutations are generated). Note that when a TR region does not begin at the first base of a codon or does not end at the last base of a codon, these partial codon sequences are not altered.

Tandem Repeats Finder is then used to find repeat stretches in each permutated TR region and a REGIONscore is calculated. Note that the repeat stretches in the permutated TR regions may not necessarily always overlap. If so, the REGIONscore is the sum of the scores of non-overlapping TR regions and/or TR stretches in the permutated TR region. The highest REGIONscore amongst all permutations and the original REGIONscore is considered to be the maximum possible REGIONscore.

TRANALYSER is an open source software. The code can be accessed at https://mattwilkins@bitbucket.org/mattwilkins/candida.git.

The above analyses were carried out on TR regions detected in CDS and introns could interrupt TR regions in the genomic sequence. We therefore identified intron-containing genes by comparing sequence lengths of CDS and genomic DNA for each ORF retrieved from the *C. albicans* genome database. In *C. albicans* introns are rare and apparently almost never interrupt TR-regions (see Results). We therefore included intron-containing genes in all further analyses of *C. albicans* TRs. 

### 2.2. Analysis of Tandem Repeats in Other Genomes

We used TRANALYSER to also analyse protein-coding sequences in the genomes of *Arabidopsis thaliana* and in the human genome. Because these genomes are large and these analyses are computationally intensive, we restricted them to a selection of genes.

For *A. thaliana* all representative gene model sequences were downloaded in April 2018 from The *Arabidopsis* Information Resource (TAIR) at https://www.arabidopsis.org/download/index-auto.jsp?dir=%2Fdownload_files%2FSequences%2FTAIR10_blastsets (TAIR10_cds_20110103_representative_gene_model_updated; TAIR10_seq_20110103_representative_gene_model_updated). 

For the human genome, a random selection of 8394 CDS and ORF sequences were downloaded from the UCSC Genome Browser in May 2018 (http://genome.ucsc.edu/cgi-bin/hgTables). To avoid low quality gene models, only genes that encoded proteins with an entry at the high quality swiss-prot database (https://web.expasy.org/docs/swiss-prot_guideline.html) were considered for selection.

The genomes of both species contain large numbers of introns. Therefore, we identified TR regions both in the genomic sequence and in the CDS of each gene. We determined for each TR region in the genomic sequence if it was identical to, or contained, a TR region present in the CDS. If so, we considered the TR region to be a pcTR region, uninterrupted by introns. Based on these analyses we calculated the total number of TR-regions in genes and the percentage of such regions that is not interrupted by introns.

For TR regions that did not overlap with introns we calculated REGIONscore and determined the maximum possible REGIONscore. In the case of *Arabidiopsis* the latter was done only for 630 TR regions in 502 TR-ORFs, selected from amongst all 9129 regions in 7115 genes. Selection was based on the ranking of REGIONscores so that the set was equally representative across the entire range of REGIONscores. 

### 2.3. Determination of Allele Distributions for Six TR Regions

To determine TR region length allele distributions, 58 strains (Table S1), were chosen [27] to best represent the genotypes of an international collection of 461 DNA fingerprinted commensal and disease-causing isolates [28,29,30,31,32,33] All of these strains had only been briefly cultured after their isolation and had subsequently been maintained as glycerol stocks at −80 °C. 

TR regions in genes *IFF6*, *HYR3*, *PGA55*, *EAP1* and *orf 19.1075* were amplified by colony PCR [17]. To do so, a portion of a *C. albicans* colony from each strain was picked with a 10 µL pipette tip and mixed with 20 µL PCR reaction mix. The mix contained the pair of primers that amplified a region (Table S2), 1 U *Taq* DNA polymerase (Qiagen Pty Ltd., Clifton Hill Vic, Australia), 4 µL of Q-buffer, 2 µL of 10 × PCR buffer supplied by the manufacturer (Qiagen), 10 pmol of each primer and 200 µM of each dNTP (Roche Diagnostics, Auckland, New Zealand). The cycling conditions, varied according to primer sets and the size of the products and included an initial incubation for 5 min at 96 °C, followed by 30 cycles of 45 s at 94 °C, 45 s at 50–60 °C and 30 s to 3 min at 72 °C. All PCR protocols included a final 5 min extension step at 72 °C. Reactions were carried out in an Eppendorf Mastercycler thermocycler (Eppendorf, Hamburg, Germany). As a positive control, the products were also amplified in laboratory strain SC5314.

The sizes of PCR products were measured on 2% agarose gels, against a 1 kb plus DNA ladder (Invitrogen Life Science Technologies, Auckland, New Zealand). Sizes of select PCR products were also determined on a 3730 Genetic Analyzer (Applied Biosystems, Waltham, MA, USA) [16] to verify visually detected size differences on gels (and that bands apparently of the same size indeed represented product of identical length). 

## 3. Results and Discussion

### 3.1. Almost Half of the ORFs in the C. albicans Genome Are TR-ORFs

The *Candida albicans* diploid genome assembly (strain SC5314, assembly 22) contains 12421 different ORFs (representing 6226 loci). We used Tandem Repeats Finder [26] to identify TR stretches in the 12421 coding sequences (CDS) from these ORFs. We identified 5708 TR-ORFs (i.e., ORFs containing one or more TR stretches) corresponding to 2936 gene loci (on some occasions TR stretches were detected only in one copy of a gene; Table S3). Tandem Repeats Finder often identifies multiple partially or wholly overlapping repeat stretches in the same region of DNA. We therefore wrote software, part of the TRANALYSER package we developed for the analyses of TR-ORFs reported here, that combined any overlapping repeats into TR regions (see Table 1 for some examples of TR regions and Table S3 for all TR regions). The 5708 TR-ORFS contained 12122 such TR regions. Introns are present in 252 TR-ORFs and these could interrupt some TR regions we had discovered in the CDS.’ However, of 31 TR regions in intron-containing TR-ORFs analysed, none was disrupted by an intron and thus such interruptions seem rare. Thus 46% of all ORFs in the *C. albicans* genome are TR-ORFs. 

Over half of TR-ORFS (2988 of 5709 TR-ORFs) contained only one TR region but up to 21 could be present in a TR-ORF (Table S3). TR region sizes varied from 20 bp to 5156 bp (median: 35 bp; average 48 ± 105 bp). The repeated amino acid motifs encoded by the repeat units ranged in length from 1 amino acid to 162 amino acids (see Table 1 for some examples of TR regions and Table S3 for all TR region-encoded amino acid regions). The combined length of all TR-regions was 590 kb, equivalent to 3% of all 18 Mb protein-coding DNA.

### 3.2. TR-ORFs Considerably Increase the Genome-Wide Frequency of Mutations in C. albicans Exons 

The mutation rate of a TR region depends on a number of characteristics, mainly the length of the repeat unit, the number of units present and their purity (the sequence similarity of the repeating units) [25]. Legendre et al. [25] developed a software (SERV) that generates a score (VARscore) for a TR region, based mainly on these characteristics. VARscores indicate how likely it is that allelic variants of a repeat region will be found when populations are assessed. While VARscores are not direct predictors of mutation rate, these authors established, in haploid *S. cerevisiae*, that mutation rate is exponentially related to VARscore [25].

SERV calculates VARscores for TR stretches which often overlap, and we combined these to calculate a REGIONscore, a VARscore for a TR region composed of a set of overlapping stretches (see Methods for details and Table 1 for examples of TR regions and their REGIONscores). 

The average REGIONscore of all pcTR regions was −0.449 (range −1.833 to 3.917) corresponding to an estimated rate of 2.7 × 10^−7^ mutations per region per generation, or 3 × 10^−3^ mutations per generation in one of the 12,221 pcTR regions in the genome, according to the relationship between VARscore and mutation rate established by Legendre et al. [25]. We had earlier measured pcTR mutation rates in *C. albicans* [16,17] for two genes (*PNG2* and *SSR1*). In both cases these were ~7 times higher than anticipated, based on their REGIONscores and the relationship between VARscore and mutation rate determined by Legendre et al. [25]. This was not unexpected because Legendre et al. had assessed mutation rates in haploid *S. cerevisiae*, whereas *C. albicans* is diploid [19]. Diploidy would be expected to increase TR mutation rates by providing opportunities for recombination between alleles [5].

Thus, our best estimate is a rate of 2 × 10^−2^ mutations per generation in one of the 12,221 pcTR regions in the *C. albicans* genome, that is, one mutation every 44 divisions. Aside from TR indels, one mutation every 184 divisions is expected to occur across the entire 18Mb of protein-coding DNA (assuming a mutation rate of non-repetitive DNA of 3 × 10^−10^ per bp [34,35]). This suggests that ~80% of all mutations that occur in protein-coding DNA in *C. albicans* are pcTR mutations. 

The strongest potential impact of mutations in pcTR regions would be frameshifts. However, approximately 85% of pcTR regions contained repeat stretches for which Tandem Repeats Finder reported units with lengths divisible by 3 (Table S3), indicating that changes in the number of repeat units will not lead to frameshifts. Tandem Repeats Finder does not report all possible unit sizes [26] and thus, for instance, three copies of a 4 bp unit may form a 12 bp unit, which may not be reported (an example, C2_02330W_B 1, is shown in Table 1). Thus, insertions and deletions of units in the remaining 15% of pcTR-regions will also often not affect the reading frame. 

Most indels in pcTR regions are thus likely to alter the number of amino acid repeats. Expansions and contraction of amino acid repeat sequences can have considerable phenotypic consequences but in many cases the effect is probably minor [1,5,9]—as is also the case with other mutations [36]. However, in microbial species, even changes that affect fitness by less than 0.0001% are under selection [11,12,13].

### 3.3. TR-ORFs Are Not Restricted to Particular Functions, Processes and Components

**Figure 2:**
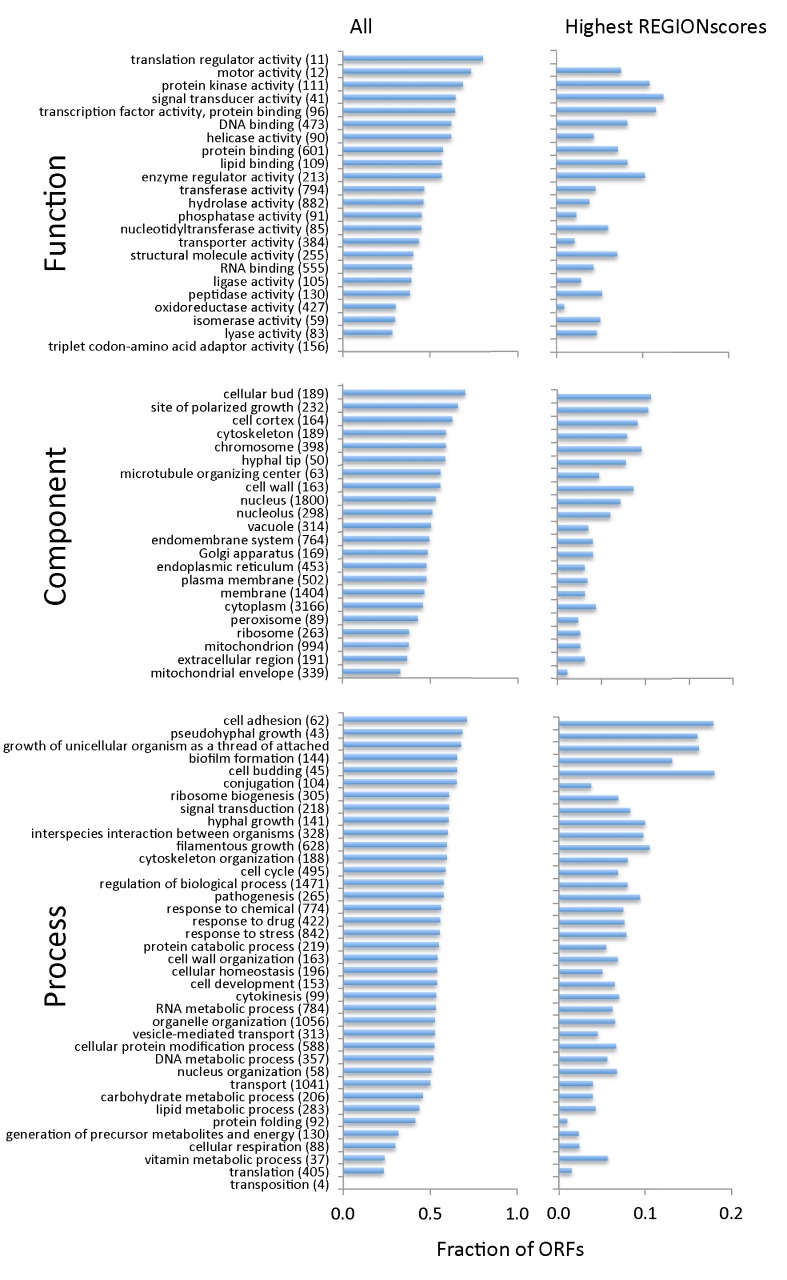
Frequency of TR-ORFs in different gene categories. Shown are the fractions of all genes in the *C. albicans* genome mapped to GO slim terms that are TR-ORFs. Numbers in brackets next to a term are the total number of genes in the genome mapped to that term. Shown on the left (All) are fractions of genes containing TR-ORFs. Fractions on the right (Highest REGIONscores) show the fraction, in each gene category, of TR-ORFS containing pcTRs whose REGIONscores are in the top 10% of all REGIONscores.

Given the estimated impact of pcTR regions on mutation rate, it was of interest to determine what types of genes contain pcTR regions. To do so we used the GO annotation of the genome [24]. Figure 2 shows what percentage of all *C. albicans* genes associated with each GO term contains pcTR-regions. We expected that proteins involved in interaction with the host, in particular with cell surface-related roles, would often be encoded by TR-ORFS as well as regulatory proteins, as has been observed in other eukaryotes [5,6,37]. This was the case but the most striking outcome of this analysis was that for all but two GO terms more than 20% of all proteins assigned to a term were encoded by TR-ORFs. We identified the top 10% pcTR regions in terms of REGIONscore and repeated the analysis with the 300 genes that contained them but even these were fairly evenly distributed between GO categories (Figure 2)—although the difference in the frequency of high-scoring pcTR region-containing genes between some Process categories (adhesion, some forms of growth and biofilm formation) and the frequencies of such genes in the remaining categories was greater than when all pcTR regions were considered. 

### 3.4. Amino Acid Repeat Units and Amino Acid Repeat Region Lengths Are under Selection and TR Mutation Generates Clade-Specific Protein Variants

A number of lines of evidence support the notion that pcTR regions are under selection in *C. albicans*, albeit to date only based on a small percentage of all pcTRs. These include (i) direct demonstration of a functional role of the pcTR region [38,39,40,41,42] (ii) functional impact of altered pcTR region length [43,44,45,46] (iii) elevated ratios of synonymous to nonsynonymous mutations in repeat units [47] and (iv) non-random distribution within pcTR regions of repeat units with nonsynonymous mutations [16,17]. 

The species *C. albicans* is subdivided into clades that diverged 3–16 million years ago [48] and additional evidence that pcTR regions are under selection is the existence of clade-specific TR-ORF alleles or allele combinations—i.e., for a given pcTR-containing locus strains belonging to the same clade will often have the same combination of two pcTR region lengths [16,17,43,47,49,50,51]. Given that a large number of alleles and allele combinations exist for these TR-ORFs [16,17,43,47,49,50,51], the rate of pcTR mutation and the time that has elapsed since clades diverged, the prevalence of one or two allele combinations in a clade is difficult to explain except by selection [16,17,52]; we have calculated that a few thousand years would have sufficed to eliminate clade-specificity of TR region length allele combinations if these were selectively neutral [17].

Clade-specific TR-ORFs and TR-ORF genotypes differing in pcTR lengths also offer an evolutionary explanation for the high frequency of pcTRs in *C. albicans* genes. Namely, pcTRs would have allowed *C. albicans* to evolve protein variants and variant combinations optimized for different genetic backgrounds (including epistatic interactions of clade-optimized alleles of a given TR-ORF with others). Such fine-tuning of over 10,000 proteins may have been difficult to achieve except through highly mutable pcTRs.

Optimizing proteins for a given genetic background by incorporating highly mutable regions into the ORFs does have a significant drawback though: continuing frequent mutation of the TR region will continue to produce new protein variants, suboptimal in this genetic background. The extent of the resulting disadvantage and the fitness advantage associated with having the “correct” clade–specific version can be estimated from the frequency of “incorrect” versions. This is possible because the frequency of allele combinations that differ from the optimal pcTR region lengths should be determined by the balance between the rate at which such allele combinations arise (i.e., the mutation rate of a TR region) and the rate at which they are removed by selection because their carriers are less fit (i.e., the selection coefficient [53]; genetic drift would not be expected to play a major role in determining their frequency given the large effective population size of microbial species [11,12,13]). 

We have determined [16,17] for two TR-ORFs, *PNG2* and *SSR1*, that insertions and deletions of repeat units alter pcTR region lengths at a rate of 1.4 and 6 × 10^−5^ per generation. We have also determined that ~20% of isolates in the largest and genetically most homogeneous *C. albicans* clade 1 [52,54] have allele combinations that differ from the predominant allele combinations. Thus, assuming that the unusual allele combinations are suboptimal (at least most of the time; see below), the selection coefficients of TR region length-altering changes for these two genes can be calculated as 3 × 10^−4^ and 7 × 10^−5^, respectively. 

Assuming a frequency of ~20% of suboptimal allele combinations across all 2936 TR-ORF loci in the genome and similar selection coefficients, pcTR mutation could reduce fitness by 20–80%—a considerable reduction. However, to put these numbers in perspective, they are equivalent to the typical fitness cost of a small number of deleterious point mutations [55,56].

### 3.5. Synonymous Mutations Reduce the Repetitiveness of Many TR Regions 

Synonymous mutations can reduce the repetitiveness and thus the mutation rate of DNA encoding an amino acid repeat. For example, the amino acid repeat SSSSSSSSSSSS, encoded, using only the codon TCC, as TCC TCC TCC TCC TCC TCC TCC TCC TCC TCC TCC TCC has a VARscore of 1.04. In contrast, the sequence TCC TCA TCT TCA TCT TCC TCC TCA TCT TCA TCT TCC encoding the same amino acid repeat but using three different codons, has a VARscore of −0.26, equivalent to a predicted mutation rate 10 times lower [25] than the first sequence.

Since it is the amino acid repeat, rather than the DNA repeat that is under selection, one way to reduce the frequency at which suboptimal TR region alleles arise and thus the fitness cost associated with TR mutation, would be synonymous mutations. Even if pcTR regions initially had had no synonymous mutations, there would have been opportunity to acquire these during the evolutionary history of extant strains, judging by the frequency of point mutations present in their genomes: Bougnoux et al. found one polymorphic site every 34 bases in a set of 92 isolates [57] and the SC5314 genome contains one single nucleotide polymorphism (SNP) every 83 base pairs [58]. 

If reduction of the repetitiveness of a TR region encoding an amino acid repeat is selectively advantageous, synonymous mutations would be retained after they arose. In addition, the repeat unit that contained them would “spread” replacing other units by recombination and replication slippage events to further reduce repetitiveness of the region. Synonymous mutations can also carry a fitness cost, in the form of reduced efficiency of translation if the codon is rarely used [59]. However, given the abundance of TR regions and given that the age of many TR regions presumably exceeds the divergence of clades (as indicated by the existence of clade-specific alleles [16,17,43,47,49,50,51]), codon usage patterns should have co adapted if *C. albicans* had engaged in a strategy of using synonymous mutations to reduce the repetitiveness of pcTR regions. 

We incorporated into TRANALYZER a feature that allowed us to assess if *C. albicans* uses synonymous mutations to reduce the repetitiveness of TR regions without altering the amino acid repeat. To do so, TRANALYZER eliminates and shuffles synonymous mutations in TR regions (see Methods and Figure 1b). It then recalculates the REGIONscores of these permutations in an attempt to find the highest REGIONscore. 

**Figure 3:**
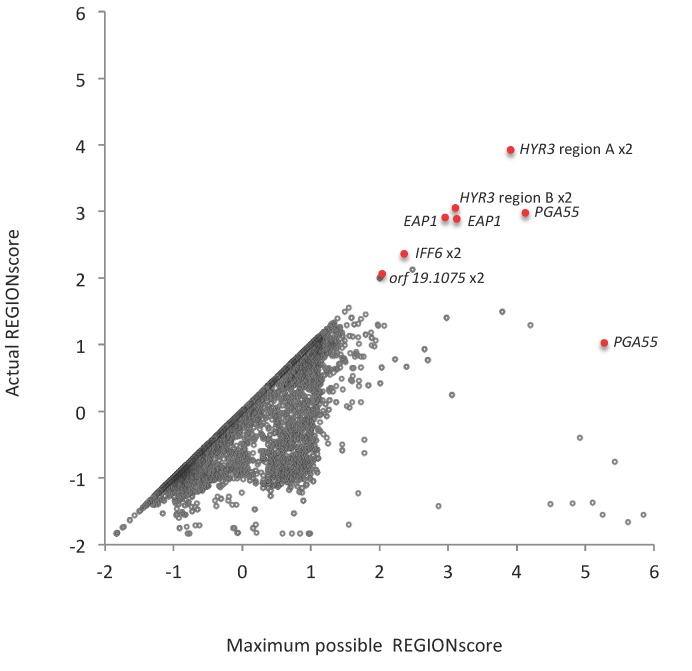
Plot of actual REGIONscores against highest possible REGIONscores for all *C. albicans* pcTR regions. pcTR regions with REGIONscores >2 for which allele distributions were investigated in a strain survey are marked in red and labelled. “×2” indicates that the data point represents the values for both alleles in the SC5314 genome.

Using this methodology, we found REGIONscores of theoretical permuted sequences that were higher than the observed scores for the same regions for 5220 of the 12,122 TR regions (Figure 3). The average reduction of REGIONscores by synonymous mutations in these 5220 TR regions was 0.16. The average REGIONscore across all 12,122 TR regions was reduced by 0.21 through synonymous mutations, from a maximum possible average REGIONscore of −0.25 to its actual value of −0.45. This correspond to a 25% reduction in the estimated genome-wide TR mutation rate [25].

Thus *C. albicans* widely uses synonymous mutations to reduce the repetitiveness and thus the mutation rate of pcTR regions.

**Figure 4:**
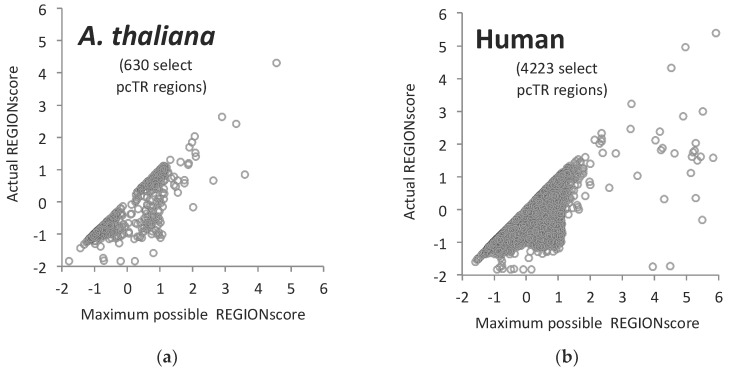
Plot of actual REGIONscores against highest possible REGIONscores for TR regions in (**a**) *A. thaliana* and (**b**) *Homo sapiens.* Values for a small selection of pcTR regions are shown.

It seemed of interest to explore if reduction of TR mutation rate by synonymous mutations might be a mechanism employed by eukaryotes in general. We therefore carried out the same type of analysis in two unrelated eukaryote species, in the plant *Arabidopsis thaliana* and in humans. We found that both of these species reduce repetitiveness of pcTR regions by synonymous mutations to a similar degree as *C. albicans* (Figure 4). 

We noticed that in these intron-rich genomes the location of pcTR regions relative to introns may be an additional mechanism for altering their mutation rates. In *A. thaliana* 50% of genes contained TR regions in their transcribed regions but only 40% of these TR regions were identical to, or contained, TR regions present in the CDS. In the human genome 90% of ORFs we analysed contained TR regions but only 1.6% of these TR regions were identical to, or contained, TR-regions present in the CDS (although this fraction was strongly size-dependent; in CDS’ of 3–6 kb, for instance, 20.1% were not interrupted by introns and in CDS > 400 kb 0.2%). 

### 3.6. TR-ORFS with Close-to-Maximal REGION Scores. Are They Contingency Genes?

Clades seem well adapted to the range of habitats *C. albicans* is likely to encounter in the human host, apparently its primary habitats [19,20]. Within a clade, clade-specific alleles appear to be prevalent for the vast majority of loci, regardless of body location and type of individual the strains are isolated from [16,17,43,47,49,50,51,52,60]. The available evidence suggests only limited specialization of clades towards particular types of individuals, body locations or interaction with the host (pathogenic versus commensal) [33,60,61]. Genetically indistinguishable strains can often be found in different body sites of an individual [29,62] and opportunities for generating specialist lineages by parasexual recombination are exceedingly rare [22,63]. All of this indicates that selection, including selection of pcTR region alleles, generally favours broadly adapted general-purpose genotypes [33] and that the clades represent different evolutionary paths towards such genotypes.

However, even if long-term success depends on maintaining a general-purpose genotype, transient reversible alterations can assist in adaptation to particular habitats [64,65]. Given their high mutation rates and the large size of populations of *C. albicans* in the human host [19] TR-ORFs would offer *C. albicans* an opportunity to slightly and transiently, alter its genotype. From the constantly replenished pool of TR-ORF variants, some might be favoured by selection in a particular habitat. These may, for a time, become the predominant variant in that habitat, later to be replaced, from the pool, by the original general-purpose allele.

In addition, there could be TR-ORFs for which no generally optimal alleles exist, including perhaps some for which fitness benefits arise from constant change: Contingency genes which put the population in a state to exploit transient habitats and to evade host responses [8,18,66,67,68]. These could for instance generate, through TR mutations, habitat-specific adhesins [39,43,44,69] or alterations of the wall [16,46,47,50,70,71]. The latter could affect host recognition [72] similar to the mechanism used by *Plasmodium falciparum* to elude the host response [73]. Provided that the ability to cause disease on occasions, or survival of the host response triggered by causing disease, is selectively sufficiently advantageous [52,74], some TR-contingency genes could have evolved to have roles in candidiasis, that is, when *C. albicans*’ interaction with the host changes from commensal to pathogenic. 

If alternative alleles of a TR-ORF assist in short-term adaptation then it is important to maintain a pool of such alleles by mutation. Thus, in such TR-ORFs mutation should have greater net benefits than in other TR-ORFs. A high REGIONscore and codon usage that maximizes REGIONscore could thus be an indication that mutation of a pcTR region is used for transient adaptation or evasion of host responses and that the TR-ORF that contains the region is a contingency gene. We identified 19 pcTR regions that on this basis could be considered most likely to have a role in short-term adaptation. They are the pcTRs with the highest REGIONscores, all ≥2, largely undiminished by synonymous mutations (Figure 3; Table S3). 

The19 pcTR regions were located in nine different genes (Table S3). Interestingly, for one of them, C2_08980C (*PGA 55*), the repeat region of only one copy of the gene is part of the set. In the same region of the second copy the actual REGIONscore is reduced to 1.0 (from a maximum score of 5.3 achievable if only one type of codon per amino acid were used; Figure 3). Also, several of the nine TR-ORFs contained additional pcTR regions with REGIONscores significantly reduced by synonymous mutations (Table S3). 

Four of the nine genes are GO–annotated as encoding proteins located on the cell surface; a 14-fold overrepresentation (GO Finder *p* = 0.001) Five are annotated as encoding adhesins or putative adhesins, far in excess of the genome-wide frequency of genes GO-annotated as involved in adhesion (“Cell adhesion”; 1%; however, while adhesion is described as their likely role, only one of them is actually formally linked to this GO term). The TR regions of two of the genes (*IFF6* and *HYR3*) encode very similar amino acid repeats and the proteins have 38% sequence identity (E value of 3 × 10^−102^ in a Blast P search against all predicted proteins in the SC5314 genome assembly).

**Figure 5:**
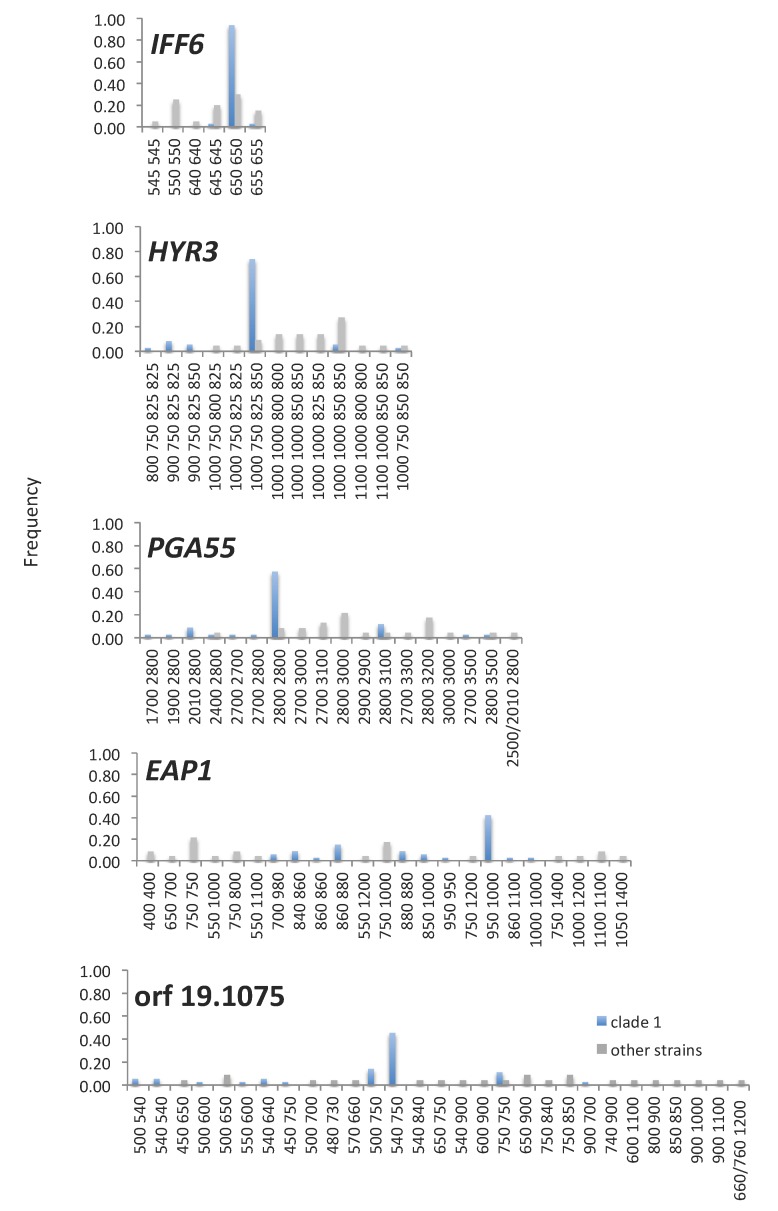
Allele combination frequencies of TR regions with high VARscores in five *C. albicans* genes in clade 1 strains (blue bars) and other strains (grey bars). Numbers indicate the size of PCR products amplified from the gene. In cases in which only one product size was obtained it was assumed that the strain had two alleles with identical TR region lengths. For *HYR* 3 allele types include both TRregion A (first pair of numbers) and TR region B (second pair of numbers).

We screened five of the genes for TR region length polymorphisms in a collection of 58 strains, both commensal and disease-causing, representative of the different clades and isolated from different body sites (Table S1; Figure 5). Because it is difficult to assemble long DNA repeat regions by sequencing, we did so by gel electrophoresis. A drawback of this methodology is that alleles of the same length cannot further be differentiated based on sequence differences and that small difference in size between alleles could be missed. Nevertheless, it can provide some insights into selective restrains of repeat region lengths. 

Genetic background did apparently play a role in selection of region lengths even in these highly mutable pcTR-regions. We assessed this by comparing lengths in 35 strains in the largest and genetically homogenous clade, clade 1 [33,61] with those in 23 strains from the remainder of the species. For all pcTR regions, one region length combination genotype was significantly more frequent in clade 1 strains compared to other strains (Fisher’s exact test, *p* values of 0.006–1.5 × 10^−6^; Figure 5; Table S1).

The number of different alleles and genotypes observable differed considerable between the pcTR-regions. Most restricted in this regard was the pcTR region in C2_09130C (*IFF6*): 70% of all alleles we assessed were of the same apparent size and only six *IFF6* genotypes were distinguishable. Also, notably, we never saw two different allele sizes in the same strain. The *HYR3* region B, encoding a series of very similar amino acid repeats, seemed under similar restraints as was the *HYR3* region A. All three regions not only had a limited number of alleles but the different alleles were also fairly similar in size.

A possible explanation of these restraints in spite of high REGIONscores undiminished by codon usage could be that the observed alleles are strongly selected under most circumstances but that there are occasions where the existence of alternate alleles in the population is crucial to survival, selecting for high mutation rates. We found some evidence supporting this hypothesis when comparing clade 1 isolates from different sources, including the bloodstream, an environment rarely encountered by *C. albicans* [75]. Of eight clade 1 bloodstream isolates, three were homozygous for an 825 bp pcTR region 2 allele, compared to one out of 27 isolates from other sites (*p* = 0.03; Fisher’s exact test).

The pcTR region of C2_08980C (*PGA55*), another likely adhesin, had a somewhat wider range of allele sizes. However, the diversity of length-based genotypes, 18 types, was comparable to that of other TR-ORFs with much lower REGIONscores (*SSR1*; 41 in 131 strains and *PNG2*; 23 alleles in 80 strains [16,17]). Thus, the *PGA55* pcTR region lengths, since predicted to be more mutable, may be under more selective constrains than those of the aforementioned genes with lower REGIONscores. 

When considering the contrast between the observed diversity of alleles and the expected diversity of these pcTR regions, based on their REGIONscores, it must be borne in mind that REGIONscores only provide estimates and cannot exactly predict how frequently indels generate new alleles. However, all of the above pcTR regions are exceptionally long (631–2283 bp; Table S3) and thus expected to be exceptionally mutable.

Length polymorphisms in the pcTR region in the adhesin *EAP1* (C2_09530W) and in the other remaining pcTR region, in C2_09130C (an uncharacterized ORF), are most in line with expectations for those of contingency genes frequently involved in short term adaptation to transient habitats, namely a wide range of allele sizes and a comparatively low frequency of the prevalent genotype. For *EAP1* we also found some indication of possible roles in habitat-specific adaptation within clade 1. The frequency of alleles sized 840–880 bp was correlated with the habitats from which strains had been obtained. They were present in all of seven bloodstream isolates, in seven of 13 commensal isolates but in only one of 13 isolates from superficial infections (Chi square test, *p* < 0.01).

## 4. Conclusions

Our data support the notion that in *C. albicans* highly mutable pcTRs allow the evolution of protein variants with properties sufficiently distinct to be under selection. This has generated protein variants that are optimized for specific genetic backgrounds. However, the high mutation rates of pcTRs are likely to represent a considerable ongoing fitness cost. *C. albicans* apparently reduces this cost by “down-regulating” the mutation rates through incorporation of synonymous mutations and does so to different degrees in different TR-ORFs. 

Even among TR-ORFs with high REGIONscores, largely undiminished by synonymous mutations we did not find any for which habitat is a stronger driver of selection than the genetic background. Thus, apparently even the most mutable pcTRs encode clade-specific protein variants optimized for performance across many habitats. This is perhaps not unexpected, given that *C. albicans*’ almost exclusively clonal mode of propagation [22] and a dynamic human *C. albicans* microbiome [62] should favour generally adapted genotypes over specialists. 

Our small survey reported here and earlier work [16] suggests that, superimposed on this genetic background-driven selection, some additional selection aimed at adaptation to specific habitats can occur. One of the strongest indications is the contrast between high REGIONscores and a very limited number of alleles in our surveys of *IFF6* and *HYR3.* It suggests that under yet-to-be identified circumstances the existence of alternate alleles is crucial. For *HYR3* and some other TR-ORFs our (very limited) information on the origin of isolates suggests that the transition to pathogenesis might be one situation that selects for alleles different from those optimal in commensalism. However, given that *C. albicans* causes disease so rarely, maintaining a pool of alternate alleles is probably more important in other situations. The colonization of the newborn from its mother [76] would be a likely one. Age-dependent changes in clade prevalence [60] suggest that the genotypes optimal for colonization of a newborn are different from those optimal for colonization of its mother. The ability to increase the chances of colonization of a new host should constitute a powerful selective pressure for maintaining a high mutation rate in a pcTR region. Further studies on additional TR regions and additional strains with detailed information regarding the hosts and the sites from which they were isolated are needed to elucidate to what degree TR mutation assists in habitat-specific adaptation.

The Red Queen hypothesis states that organisms must constantly evolve to keep up with their competitors, one of the reasons why achieving the lowest possible mutation rate is not necessarily advantageous [77,78]. Aside from offering an opportunity for optimizing alleles, introduction of pcTRs may be selectively advantageous, in spite of the constant cost of generation of suboptimal alleles, by maintaining a mutation rate sufficiently high for competition in the Red Queen’s race. This may be another major reason for the high frequency of pcTRs in the *C. albicans* genome.

One considerable advantage of pcTRs over adjusting mutation rates by altering of DNA repair and proofreading mechanisms [78] is that through the incorporation or removal of synonymous mutations the mutation rate can be adjusted for individual pcTR regions. *C. albicans* is apparently not unique among eukaryotes in employing this strategy. When we analysed the genome of *Arabidopsis thaliana* and the human genome we found that both of these species reduce repetitiveness of pcTR regions by synonymous mutations to a similar degree as *C. albicans* (Figure 4). In such intron-rich genomes the location of TR regions relative to introns may contribute additional facets to the evolutionary biology of TR regions. Interruption of amino acid repeat-encoding DNA by introns could reduce the rate at which new protein variants arise, while TR region expansion within introns can generate new protein variants, in addition to a raft of other effects [79,80]. Given the high percentage of TR regions in untranslated parts of transcribed DNA, in many genomes TR mutation may exert much of its phenotypic effect by altering introns and 5′ and 3′ untranslated regions [79,80,81,82].

Current attempts to understand the evolutionary forces that determine mutation rate are largely focused on point mutations [78]. Our results for *C. albicans* add to the growing evidence that pcTR mutation may deserve more consideration as a driver of mutational advance. 

## Figures and Tables

**Table 1:** Examples of TR region.

TR Region ^1^	REGION Score	TR Unit Lengths (bp) ^2^	Nucleic Acid and Amino Acid Sequence of Regions ^3^
C2_10280C_B 3 (*SPT23*)	1.01	3	ACAACAACAACAACAACAACAACAACAACAACAACAACAGCAGCAGCAACAGCAACAACAGCAA
			QQQQQQQQQQQQQQQQQQQQQ
C1_01560W_B 1 (*SIZ1*)	0.92	6, 3, 12, 24	ACAGCAACAGCAACAGCGACAACTTCGACAACTAGAACAGCAGCAACGGCTACAGCGACAGCAATGGCAACAGCAACAGCAACAACTACAGCAACAACAACAACAACTTCCTCGCCAACTTCCCCAACAGTCTCCCCGACAACTTCCCCAACAG
			QQQQQRQLRQLEQQQRLQRQQWQQQQQQLQQQQQQLPRQLPQQSPRQLPQQ
C2_02330W_B 1	0.28	4	CACTTACTCACTCACTCACTCACTCACTC
			LTHSLTHSL
C1_09690W_B 2	−1.15	12	TTTCTTGCCAGATTTCTTACCAGA
(*MLS1*)			FLPDFLP

^1^ Name of ORF in the *Candida* genome database followed by the number of the TR region. For ORFs of named genes this is followed by the name of the gene, in brackets. ^2^ Lengths of repeat units as reported by TR finder. ^3^ The amino acid sequences do not include a translation of partial codons at the beginning of end of a TR region.

## Supplementary Materials

The following are available online at http://www.mdpi.com/2309-608X/4/3/78/s1, Table S1: Strains and Alleles, Table S2: Primers, Table S3: REGIONscores.
