# Supplementary material for: Biological Roles of Protein-Coding Tandem Repeats in the Yeast Candida Albicans
Source: J Fungi (Basel). 2018 Jun 29;4(3):78. doi: 10.3390/jof4030078 (PMC6162428; doi:10.3390/jof4030078)
Supplement: Supplementary file 1 [file jof-04-00078-s001.zip › Supplemental Material archive final /Table S2 Primers 7May.docx]

**Table S2. PCR Primers**

| **Target TR region** | **Primer name** | **Primer sequence^1^** |
| --- | --- | --- |
| *HYR3* region A | HYR3r1pf | 5' AGTAGTTCTGAAACTGTTGC 3' |
|  | HYR3r1pr | 5' ATCGGAGTTGGTGGTAGTCC 3' |
| *HYR3* region B | HYR3r2pf | 5' ACCTCAGCTACCCAAACTGT 3' |
|  | HYR3r2pr | 5' GCCTTCGTTGGATCCATTGC 3' |
| *PGA55* | PGA55rpf | 5' TCATCTTCATCCAGTAGTAC 3' |
|  | PGA55rpr | 5' ATGACAATTCCAGCAGTAGT 3' |
| *IFF6* | IFF6rpf | 5' CCGCTACTAATACTGATAACG 3' |
|  | IFF6rpr | 5' CCATTATCTTGTCCACTACC 3' |
| *EAP1* | EAP1rpf | 5' ACCATCTCATCTGAAGAAAC 3' |
|  | EAP1rpr: | 5' CAGCTAGCGACAATTGAAGT 3' |
| *orf19.1725* | orf19.1725rpf | 5' ACTGTTGCTACTATTCCACC 3' |
|  | orf19.1725rpr | 5' GATAACATTAGTACCTGGAGT 3' |

^1^M13 tail 5' - GACGTTGTAAAACGACGGCC - 3' was added to each forward primers 5’ ends for genotyping.
